# Gender-based differences in harm reduction practices among people who use drugs in Rhode Island: a latent class analysis

**DOI:** 10.1186/s12954-025-01295-9

**Published:** 2025-08-18

**Authors:** Leah C. Shaw, Anusha Kumar, Carolyn J. Park, Yu Li, Catherine A. Lenox, Alexandra B. Collins, Susan G. Sherman, Brandon D. L. Marshall, Alexandria Macmadu

**Affiliations:** 1 Department of Epidemiology, Brown University School of Public Health, 121 South Main Street, Providence, RI 02912 USA; 2 Department of Biostatistics, Brown University School of Public Health, 121 South Main Street, Providence, RI USA; 3 Department of Community Health, Tufts University, 574 Boston Avenue, Suite 208, Medford, MA USA; 4 Department of Health, Behavior and Society, Johns Hopkins Bloomberg School of Public Health, 624 North Broadway, Baltimore, MD USA

**Keywords:** Gender characteristics, Harm reduction, Drug overdose, Latent class models, Naloxone

## Abstract

**Background:**

Previous research has documented differing drug use patterns and risk behaviors by gender identity and sex at birth, although variations in harm reduction practices by these characteristics have not yet been fully assessed.

**Methods:**

We utilized data from the Rhode Island Prescription and Illicit Drug Study (RAPIDS), which enrolled adults who used drugs from 2020 to 2023. Participants were analyzed based on gender identity: men, women, and other (including those identifying as non-binary or something else). Participants reported which harm reduction practices (e.g., use fentanyl test strips, keep naloxone nearby) they typically engaged in to avoid accidental overdose. Latent class analysis (LCA) was used to identify subgroups of harm reduction practice typologies, and a correlation matrix was generated to understand dyads of typical self-reported harm reduction practices.

**Results:**

Among 503 eligible participants, 64% were men, 34% were women, and 2% were non-binary or something else (*n* = 9). Harm reduction practices were comparable between men and women, although men were less likely to keep naloxone nearby (*p* = 0.02). LCA identified three subgroups of harm reduction practice typologies (no/low, moderate, and high utilization). Group membership in latent classes did not vary by gender identity. However, those belonging to the no/low utilization subgroup were significantly more likely to have ever been incarcerated (*p* = 0.03) and to be single (*p* < 0.01). Those belonging to the high utilization group were significantly more likely to have ever witnessed an overdose, performed rescue breathing, and administered naloxone (all *p* < 0.001). Correlations showed several pairwise relationships, with ‘use fentanyl test strips’ and ‘keeping naloxone nearby’ being positively and significantly correlated (*r* = 0.33, *p* < 0.05).

**Conclusions:**

We found that harm reduction practices and group membership in latent classes were largely comparable between men and women; however, men who use drugs are significantly less likely to keep naloxone nearby. Gender-attentive strategies to increase naloxone carriage and usage among men and enhanced outreach to persons characterized by no or low harm reduction practice utilization, including people who are single and those with a history of incarceration, may be warranted to mitigate overdose risk.

**Supplementary Information:**

The online version contains supplementary material available at 10.1186/s12954-025-01295-9.

## Introduction

The overdose epidemic in the United States (US) is persistent and ever-changing. Provisional data indicate that over 107,000 overdose deaths occurred in the US in 2023 alone [1]. Over the past two decades, the rates of drug overdose deaths have increased fourfold, and in 2022, the age-adjusted rate of drug overdose deaths reached 45.6 and 19.4 in men and women, respectively [2]. Extensive prior literature, including research from the nationwide HEALing Communities Study, has documented that the overdose mortality rate among men is approximately twice as high as that of women [3–6]. While the burden of overdose deaths is heightened among men, women who use drugs experience significant social and structural inequities that exacerbate health outcomes and inform drug use practices [7–10]. Previous research has documented differing drug use patterns and risk behaviors between men and women, including men being more likely to report lifetime and past year non-medical prescription opioid use and women being more likely to share injection equipment [11–14]. 

A secondary analysis of National Survey on Drug Use and Health data found significant gender differences in lifetime non-medical prescription opioid use: 15.9% and 11.2% of men and women, respectively [11]. In this same analysis, although there were no significant differences in opioid dependence observed by gender, men were significantly more likely to engage in treatment for alcohol or drug use [11]. Overdose prevention and harm reduction programming have predominantly been implemented without consideration for gender-based differences, and therefore, may not be sufficient to address the needs of diverse groups of people who use drugs (PWUD) [8]. Although research has studied gender-based differences in substance use patterns and overdose risk [15], there is limited prior literature examining gender-based differences in harm reduction practices in the current fentanyl era [2]. Relatedly, while latent class analysis (LCA) has been used to understand self-reported patterns of drug use and nonfatal drug overdose [16, 17], subgroups of harm reduction practice typologies have not previously been characterized.

In light of these gaps, the objectives of this analysis were to (1) examine gender-based differences in specific harm reduction practices, such as using with someone else or utilizing fentanyl test strips (FTS), and (2) determine the specific utilization patterns that characterize subgroups of harm reduction practice typologies. We sought to characterize any gender-based differences in harm reduction practice utilization and identify subgroups of PWUD who may benefit from more intensive engagement and support.

## Materials and methods

### Study design and participants

The Rhode Island Prescription and Illicit Drug Study (RAPIDS) is a randomized clinical trial that aims to assess the efficacy of an FTS intervention among PWUD. Recruitment methods have been described previously; participants were recruited from September 2020 to February 2023 [18]. State residents were eligible to participate if they were: (a) aged 18 to 65; (b) able to complete an interview in English; (c) able to provide informed consent; and (d) reported any prior 30-day use of unregulated drugs.

Participants completed a standardized, interviewer-administered questionnaire at baseline and follow-up visits over 12 months to assess recent practices and patterns. An interviewer-administered approach was selected to facilitate rapport-building and enhance data quality. Given the complexity and sensitivity of some interview questions, interviewers could clarify or explain questions when needed. Moreover, given the survey length (approximately one hour), interviewer-assisted questionnaires were preferred over computer-based methods to support completeness without influencing responses. A 12-month follow-up period was chosen to capture both short-term and medium-term patterns of behavior, especially since some events of interest (i.e., overdose, incarceration) may occur less frequently. To minimize recall bias, questions were specifically asked about behaviors within a defined time period (e.g., prior month). All staff were trained to minimize social desirability and interviewer bias. The baseline questionnaire elicited sociodemographic information, drug use patterns, clinical characteristics, and harm reduction practices (i.e., using FTS, keeping naloxone nearby). Data from baseline assessment are analyzed here (*N* = 503). RAPIDS was approved by the Brown University Institutional Review Board.

### Measures

The primary outcome of interest in this analysis was self-reported harm reduction practices. To assess typical harm reduction practices at baseline, participants were asked, “What do you do to avoid an accidental overdose?” The following response options were then read aloud, with participants encouraged to select all that apply: nothing, avoid mixing with alcohol, avoid mixing with other drugs, smell or taste my supply, use with someone else, take smaller amounts, go slow, take a tester, use FTS, keep naloxone nearby, change supplier or dealer, or something else.

The primary independent variable of interest is current gender identity, which was used to stratify results. Participants were asked, “What best describes your current gender identity?” and could see the list of options. Research assistants would prompt participants by reading aloud the options if necessary. Response options included male, female, transgender male/trans man/female-to-male, transgender woman/trans woman/male-to-female, genderqueer/neither exclusively male nor female, something else, or don’t know/refused. Those who answered ‘don’t know’ or refused (*n* = 2) were excluded. In this analysis, men are defined as those identifying as either “male” or transmen, and women include those identifying as either “female” or transwomen. Those who identified as genderqueer/neither exclusively male nor female, or something else, were combined and categorized as “other gender identity.” Baseline characteristics are stratified by men and women. Other sociodemographic characteristics of interest included age and race/ethnicity (categorized as white and non-Hispanic, Black and non-Hispanic, other race(s) and non-Hispanic, and Hispanic/Latine of any race). Sexual orientation categories were operationalized to combine lesbian, gay, bisexual, and queer and compared with those who self-identified as straight. We also analyzed information on relationship status (cohabiting with a spouse/partner, not cohabiting with a spouse/partner, dating/seeing someone, single), prior month homelessness (yes, no), individual monthly take-home income (≤$500, $501–1500, >$1500; in US dollars), and lifetime incarceration (yes, no). Of note, income was categorized using response options from the original survey instrument; response options were collapsed into three groups to better reflect the distribution of responses and simplify the presentation of results.

Years since initiating injection and non-injection (i.e., inclusive of snorting, sniffing, swallowing) routes of administration were derived using self-reported age at initiation of each modality and current age reported at the baseline assessment. Past-month regular substance use was defined as at least four days of use in the prior 30 days. Participants were asked about extra-medical prescription opioids, benzodiazepines, prescription stimulants, crystal methamphetamine, crack cocaine, powder cocaine, and heroin. Regular fentanyl use was operationalized differently during baseline assessment, so it was not combined with heroin or other extra-medical opioid use. Regular use of fentanyl was defined as “at least weekly” or “every day.”

Lifetime drug use behaviors, including injection drug use (IDU) (yes, no), helping someone else inject (yes, no), sharing syringes or drug use-related supplies (yes, no), and drug selling (yes, no) were also analyzed. Both prior month (yes, no) and lifetime history (yes, no) of overdose are included, as well as ever witnessing an overdose (yes, no), ever performing rescue breathing (yes, no), and ever administering naloxone (yes, no). Clinical characteristics included current use of any opioid agonist therapy (OAT) such as methadone or buprenorphine (yes, no). Self-reported lifetime diagnoses of three mental health conditions, including depressive disorder (yes, no), anxiety disorder (yes, no), and bipolar disorder (yes, no), were also analyzed.

### Statistical analysis

We first analyzed descriptive statistics of sociodemographics, drug use patterns, clinical characteristics, and harm reduction practices for both the overall sample of PWUD and stratified by gender identity. Bivariate associations compared men and women only and were examined using Wilcoxon rank sum tests, Pearson’s chi-squared, or Fisher’s exact tests as appropriate [19, 20]. A *p*-value of < 0.05 was considered statistically significant. Those belonging to the other gender identity group (*n* = 9, 2%) were excluded from comparative analyses, given the small sample size of this subgroup.

This analysis used latent class analysis (LCA) to identify subgroups that share harm reduction practice typologies. All primary outcomes (i.e., each of the 12 harm reduction practices) were dichotomized. Standard model fit statistics were used to determine the best-fitting model, including entropy, Akaike information criterion (AIC), and Bayesian information criterion (BIC). Initial class enumeration was conducted by estimating models with increasing numbers of classes. Once class membership was assigned to each participant, the harm reduction variable (i.e., latent class) was treated as a primary outcome. Covariates of interest were then stratified by latent class. The latent class model fit statistics are presented in Supplementary Tables 1, and harm reduction practice probabilities for the final three-class model are presented in Supplementary Table 2.

Finally, we assessed correlations between dyads of harm reduction practices. A matrix was created to highlight all harm reduction practices and the most important relationships between them; Supplementary Table 3 includes all coefficients. Analyses were completed in R [21–23]. 

## Results

### Baseline characteristics

Sociodemographics, drug use patterns, clinical characteristics, and harm reduction practices of the 503 individuals who were included in this analysis are presented in Table 1. The sample included 322 men (64%), 172 women (34%), and 9 participants belonging to other gender identities (2%) (Table 1). The vast majority (96%) of the sample was cisgender, and cross-tabulations of gender identity at baseline by sex at birth are presented in Supplementary Table 4. The median age of participants was 43 years (IQR 35, 53). More than half (52%) of the sample was non-Hispanic white, 17% were non-Hispanic Black, and 21% were Hispanic/Latine of any race. The most frequently reported drugs used regularly were crack cocaine (61%), heroin (32%), fentanyl (30%), and powder cocaine (27%). More than half of the sample had lifetime experience with IDU (55%) and overdose (54%). Almost three-quarters (73%) of participants reported any mental health diagnosis, and 44% reported a diagnosis of anxiety and/or depression. Significantly more women reported these diagnoses compared to men (all *p* < 0.05), which is consistent with gender-based differences in the general population [24]. 

**Table 1:** Sociodemographics, drug use patterns, clinical characteristics, and harm reduction practices of 503 people who use drugs in Rhode Island from September 2020 to February 2023, stratified by current gender identity

Characteristics	Overall^a^ *N* = 503	Men *n* = 322 (64.0%)	Women *n* = 172 (34.2%)	*P*-value^b^
*Sociodemographics*				
Age, median (IQR)	43 (35, 53)	43 (34, 53)	43 (36, 53)	0.453
Race/ethnicity				
White and non-Hispanic	259 (51.6)	165 (51.4)	90 (52.3)	0.216
Black^c^ and non-Hispanic	83 (16.5)	51 (15.9)	32 (18.6)	
Other race(s) and non-Hispanic	56 (11.2)	30(9.3)	22 (12.8)	
Hispanic/Latine of any race	104 (20.7)	75 (23.4)	28 (16.3)	
Sexual orientation				
LGBQ	79 (15.8)	33 (10.3)	41 (23.8)	**< 0.001**
Straight	422 (84.2)	287 (89.7)	131 (76.2)	
Transgender identity				
Cisgender	484 (96.2)	321 (99.7)	163 (94.8)	**< 0.001**
Transgender/other	19 (3.8)	1 (0.3)	9 (5.2)	
Relationship status				
Single	283 (56.6)	211 (65.9)	66 (38.6)	**< 0.001**
Dating/seeing someone	57 (11.4)	33 (10.3)	24 (14.0)	
Non-cohabiting regular partner	36 (7.2)	17 (5.3)	19 (11.1)	
Cohabiting spouse/partner	124 (24.8)	59 (18.4)	62 (36.3)	
Homelessness, prior month	295 (58.6)	207 (64.3)	84 (48.8)	**< 0.001**
Monthly income				
≤ $500	226 (45.4)	157 (49.2)	67 (39.4)	0.094
$501–1500	233 (46.8)	137 (42.9)	90 (52.9)	
> $1500	39 (7.8)	25 (7.8)	13 (7.6)	
Incarcerated, ever	390 (78.0)	273 (85.3)	113 (66.1)	**< 0.001**
*Drug use patterns*				
Years since non-IDU initiation, median (IQR)^d^	26 (16, 35)	26 (17, 36)	24 (16, 33)	0.390
Years since IDU initiation, median (IQR)^e^	13 (7, 25)	13 (6, 26)	12 (7, 23)	0.934
Substances used regularly				
Prescription opioids^f^	121 (24.1)	84 (26.1)	35 (20.3)	0.155
Benzodiazepines^f^	109 (21.7)	73 (22.7)	34 (19.8)	0.455
Extra-medical prescription stimulants^f^	64 (12.7)	45 (14.0)	16 (9.3)	0.133
Crystal methamphetamine^f^	81 (16.1)	58 (18.0)	21 (12.2)	0.094
Powder cocaine^f^	135 (26.8)	95 (29.5)	39 (22.7)	0.104
Crack cocaine^f^	306 (60.8)	195 (60.6)	106 (61.6)	0.817
Heroin^f^	160 (31.8)	114 (35.4)	45 (26.2)	**0.036**
Fentanyl^g^	140 (29.7)	96 (31.9)	41 (25.2)	0.129
Injection drug use, ever	278 (55.3)	191 (59.3)	80 (46.5)	**0.006**
Assisted injection, ever	189 (37.6)	117 (36.3)	67 (39.0)	0.566
Share syringes/supplies, ever	167 (33.2)	108 (33.5)	54 (31.4)	0.629
Drug selling, ever	344 (68.9)	234 (73.6)	104 (60.5)	**0.003**
Overdose history				
Ever	270 (54.1)	179 (55.9)	86 (50.6)	0.258
Prior month	38 (7.6)	28 (8.8)	9 (5.3)	0.163
Overdose response, ever				
Witness	429 (85.6)	276 (86.3)	146 (84.9)	0.679
Perform rescue breathing	256 (50.9)	166 (51.6)	88 (51.2)	0.934
Administer naloxone	302 (60.0)	190 (59.0)	108 (62.8)	0.413
*Clinical characteristics*				
Opioid agonist therapy, current	124 (24.8)	73 (22.8)	49 (28.5)	0.164
Any mental health diagnosis	364 (72.7)	219 (68.4)	139 (80.8)	**0.003**
Depressive disorder	220 (43.7)	123 (38.2)	93 (54.1)	**< 0.001**
Anxiety disorder	220 (43.7)	119 (37.0)	96 (55.8)	**< 0.001**
Bipolar disorder	175 (34.8)	100 (31.1)	70 (40.7)	**0.032**
*Harm reduction practices*				
Avoid mixing with alcohol	112 (22.3)	76 (23.6)	31 (18.0)	0.152
Avoid mixing with other drugs	143 (28.4)	91 (28.3)	49 (28.5)	0.957
Smell or taste my supply	96 (19.1)	63 (19.6)	27 (15.7)	0.289
Using with someone else	169 (33.6)	106 (32.9)	60 (34.9)	0.660
Take smaller amounts	222 (44.1)	140 (43.5)	77 (44.8)	0.783
Go slow	199 (39.6)	131 (40.7)	62 (36.0)	0.314
Take a tester	115 (22.9)	73 (22.7)	39 (22.7)	0.999
Use fentanyl test strips	90 (17.9)	54 (16.8)	34 (19.8)	0.407
Keep naloxone nearby	217 (43.1)	127 (39.4)	86 (50.0)	**0.024**
Change supplier or dealer	87 (17.3)	55 (17.1)	30 (17.4)	0.919
Something else^h^	38 (7.6)	24 (7.5)	13 (7.6)	0.966
Nothing	63 (12.5)	47 (14.6)	16 (9.3)	0.093

a) Those who identify as another gender identity (*n* = 9) are included in the overall analysis but are not shown as a separate column in this table to protect the anonymity of these participants

b) *P*-values calculated for pairwise comparisons via the Wilcoxon Rank Sum Test for continuous variables, Chi-square test for categorical variables, and Fisher’s Exact Test for categorical variables with cell counts < 5. Bold indicates a significant *p*-value of < 0.05

c) Includes self-identified African, Haitian, and Cape Verdean ancestry

d) Non-IDU initiation is defined as the use of an unregulated drug (i.e., heroin, cocaine, methamphetamine, psychedelics, club drugs, or something else) via snorting, smoking, or swallowing

e) IDU initiation is defined as the use of an unregulated drug (i.e., heroin, cocaine, methamphetamine, psychedelics, club drugs, or something else) via injection. Participants who reported no lifetime IDU were excluded from this measure

f) Indicates at least 4 days of use in the prior 30 days

g) Indicates “at least weekly” or “every day” use frequency

h) Includes the following responses, among others: stop or avoid drug use, avoid drugs with overdose risk, use the same supplier or talk to supplier, cook or re-cook drugs, use responsibly

Overall, the most frequently reported harm reduction practices were taking smaller amounts at a time (44%), keeping naloxone nearby (43%), going slow (40%), and using with someone else (34%). Notably, 13% of participants did not report engaging in any harm reduction practices, and 8% responded that they did ‘something else.’ All self-reported harm reduction practices captured under other responses included as ‘something else’ (e.g., avoiding specific drugs, cooking or recooking drugs) are described in Supplementary Table 5.

In pairwise comparisons of men and women, several sociodemographic characteristics differed significantly: more men identified as straight and cisgender (*p* < 0.001). There was a significant association between gender and relationship status (*p* < 0.001), suggesting that more men identified as single compared to women (66% compared to 39%, respectively). More men were also homeless in the prior month and had lifetime experience with incarceration (both *p* < 0.001). Drug use was similar between men and women. However, more men had lifetime experience with IDU (*p* = 0.006) and drug selling (*p* = 0.003), and a greater proportion of men reported using heroin regularly (*p* = 0.036). Harm reduction practices were broadly similar between men and women, although significantly fewer men reported keeping naloxone nearby (*p* = 0.024).

### Latent class estimation

Latent class models were compared to determine the optimal model to characterize typologies of harm reduction practices. The BIC statistic favored a three-class solution (Supplementary Table 1), and each of the three-class model latent classes was interpretable, sufficiently sized, and well-defined: no/low, moderate, and high utilization of harm reduction practices. Most participants who did not report engaging in any harm reduction practices were assigned to the no/low utilization group. Those in the moderate utilization class were characterized by significantly greater use of other, undefined harm reduction practices (i.e., endorsing “something else”). The probabilities of class assignment across each of the 12 harm reduction practices are presented in Supplementary Table 2. There was no missingness in LCA estimation.

Sociodemographics, drug use patterns, and clinical characteristics by latent class categories are presented in Table 2; harm reduction practices by latent class categories are presented in Table 3. The largest proportion of the sample was assigned to the moderate utilization group (61%), followed by the high utilization group (27%) and the no/low utilization group (12%). Importantly, there were no significant differences in harm reduction utilization class by gender, age, race/ethnicity, sexual orientation, or recent homelessness.

**Table 2:** Sociodemographics, drug use patterns, and clinical characteristics of 503 people who use drugs in Rhode Island from September 2020 to February 2023, stratified by harm reduction latent class category

Characteristics	Latent class category			*P*-value^a^
	No/low utilization *n* = 62 (12.3%)	Moderate utilization *n* = 307 (61.0%)	High utilization *n* = 134 (26.6%)	
*Sociodemographics*				
Gender identity				
Men	46 (74.2)	193 (62.9)	83 (61.9)	0.175
Women	16 (25.8)	110 (35.8)	46 (34.3)	
Other^b^	0 (0.0)	4 (1.3)	5 (3.7)	
Age, median (IQR)	43.0 (18)	44.0 (19)	39.5 (17)	0.057
Race/ethnicity				
White and non-Hispanic	33 (53.2)	147 (48.0)	79 (59.0)	0.066
Black^c^ and non-Hispanic	11 (17.7)	61 (19.9)	11 (8.2)	
Other race(s) and non-Hispanic	7 (11.3)	37 (12.1)	12 (9.0)	
Hispanic/Latine of any race	11 (17.3)	61 (19.9)	32 (23.9)	
Sexual orientation				
LGBQ	7 (11.3)	52 (17.0)	20 (14.9)	0.500
Straight	55 (88.7)	253 (83.0)	114 (85.1)	
Gender identity				
Cisgender	60 (96.8)	295 (96.1)	129 (96.3)	1.00
Transgender/other	2 (3.2)	12 (3.9)	5 (3.7)	
Relationship status				
Single	46 (74.2)	166 (54.6)	71 (53.0)	**0.003**
Dating/seeing someone	3 (4.8)	37 (12.2)	17 (12.7)	
Non-cohabiting regular partner	1 (1.6)	31 (10.2)	4 (3.0)	
Cohabiting spouse/partner	12 (19.4)	70 (23.0)	42 (31.3)	
Homelessness, prior month				
No	28 (45.2)	127 (41.4)	53 (39.6)	0.760
Yes	34 (54.8)	180 (58.6)	81 (60.4)	
Monthly income				
≤ $500	27 (43.5)	128 (42.4)	71 (53.0)	0.205
$501–1500	29 (46.8)	147 (48.7)	57 (42.5)	
> $1500	6 (9.7)	27 (8.9)	6 (4.5)	
Incarcerated, ever				
No	6 (9.8)	69 (22.5)	35 (26.3)	**0.034**
Yes	55 (90.2)	237 (77.5)	98 (73.7)	
*Drug use patterns*				
Years since non-IDU initiation, median (IQR)^d^	28 (19, 37)	27 (17, 36)	22 (14, 31)	**0.011**
Years since IDU initiation, median (IQR)^e^	16 (6, 20)	15 (7, 26)	11 (6, 25)	0.209
Substances used regularly				
Prescription opioids^f^	11 (17.7)	71 (23.1)	39 (29.1)	0.186
Benzodiazepines^f^	8 (12.9)	72 (23.5)	29 (21.6)	0.184
Extra-medical prescription stimulants^f^	3 (4.8)	44 (14.3)	17 (12.7)	0.108
Crystal methamphetamine^f^	7 (11.3)	45 (14.7)	29 (21.6)	0.101
Powder cocaine^f^	19 (30.6)	71 (23.1)	45 (33.6)	0.057
Crack cocaine^f^	33 (53.2)	182 (59.3)	91 (67.9)	0.099
Heroin^f^	15 (24.2)	92 (30.0)	53 (39.6)	**0.054**
Fentanyl^g^	14 (25.5)	79 (27.3)	47 (37.0)	0.106
Injection drug use, ever	29 (46.8)	159 (51.8)	90 (67.2)	**0.004**
Assisted injection, ever	20 (32.3)	110 (35.8)	59 (44.0)	0.172
Share syringes/supplies, ever	17 (27.4)	102 (33.2)	48 (35.8)	0.509
Drug selling, ever	38 (62.3)	214 (70.2)	92 (69.2)	0.478
Overdose history				
Ever	29 (46.8)	160 (52.6)	81 (60.9)	0.130
Prior month	4 (6.5)	24 (7.9)	10 (7.5)	1.00
Overdose response, ever				
Witness	44 (72.1)	262 (85.3)	123 (92.5)	**< 0.001**
Perform rescue breathing	16 (25.8)	153 (49.8)	87 (64.9)	**< 0.001**
Administer naloxone	27 (43.5)	177 (57.7)	98 (73.1)	**< 0.001**
*Clinical characteristics*				
Opioid agonist therapy, current	12 (19.7)	68 (22.2)	44 (32.8)	**0.037**
Any mental health diagnosis	43 (69.4)	219 (71.6)	102 (76.7)	0.447
Depressive disorder	23 (37.1)	130 (42.3)	67 (50.0)	0.175
Anxiety disorder	24 (38.7)	124 (40.4)	72 (53.7)	**0.024**
Bipolar disorder	21 (33.9)	109 (35.5)	45 (33.6)	0.915

Those who identify as another gender identity (*n* = 9) are included in the latent class analysis

a) *P*-values calculated via the Kruskal-Wallis rank sum test for continuous variables, Chi-square test for categorical variables, and Fisher’s Exact Test for categorical variables with cell counts < 5. Bold indicates a significant *p*-value of < 0.05

b) Denotes other gender identity, which includes those who reported identifying as genderqueer (*n* = 3) or something else (*n* = 6)

c) Includes self-identified African, Haitian, and Cape Verdean ancestry

d) Non-IDU initiation is defined as the use of an unregulated drug (i.e., heroin, cocaine, methamphetamine, psychedelics, club drugs, or something else) via snorting, smoking, or swallowing

e) IDU initiation is defined as the use of an unregulated drug (i.e., heroin, cocaine, methamphetamine, psychedelics, club drugs, or something else) via injection. Participants who reported no lifetime IDU were excluded from this measure

f) Indicates at least 4 days of use in the prior 30 days

g) Indicates “at least weekly” or “every day” use frequency

**Table 3:** Harm reduction practices of 503 people who use drugs in Rhode Island from September 2020 to February 2023, stratified by harm reduction latent class category

Harm reduction practices	Latent class category			
	No/low utilization, *n* = 62 (12.3%)	Moderate utilization, *n* = 307 (61.0%)	High utilization *n* = 134, (26.6%)	*P*-value^a^
Avoid mixing with alcohol	0 (0.0)	34 (11.1)	78 (58.2)	**< 0.001**
Avoid mixing with other drugs	0 (0.0)	49 (16.0)	94 (70.1)	**< 0.001**
Smell or taste my supply	0 (0.0)	23 (7.5)	73 (54.5)	**< 0.001**
Using with someone else	0 (0.0)	70 (22.8)	99 (73.9)	**< 0.001**
Take smaller amounts	0 (0.0)	108 (35.2)	114 (85.1)	**< 0.001**
Go slow	1 (1.6)	89 (29.0)	109 (81.3)	**< 0.001**
Take a tester	0 (0.0)	53 (17.3)	62 (46.3)	**< 0.001**
Use fentanyl test strips	0 (0.0)	44 (14.3)	46 (34.3)	**< 0.001**
Keep naloxone nearby	1 (1.6)	97 (31.6)	119 (88.8)	**< 0.001**
Change supplier or dealer	1 (1.6)	39 (12.7)	47 (35.1)	**< 0.001**
Something else^b^	0 (0.0)	33 (10.7)	5 (3.7)	**0.001**
Nothing	62 (100)	0 (0.0)	1 (0.7)	**< 0.001**

a) *P*-values calculated via the Kruskal-Wallis rank sum test for continuous variables, Chi-square test for categorical variables, and Fisher’s Exact Test for categorical variables with cell counts < 5. Bold indicates a significant *p*-value of < 0.05

b) Includes the following responses, among others: stop or avoid drug use, avoid drugs with overdose risk, use the same supplier or talk to supplier, cook or re-cook drugs, use responsibly

Nonetheless, several sociodemographic characteristics and drug use patterns differed significantly across latent classes. More people belonging to the no/low utilization group were single (*p* < 0.001) and had a lifetime history of incarceration (*p* = 0.03). More people belonging to the high utilization group had reported lifetime IDU (*p* = 0.01), had performed rescue breathing (*p* = 0.001), and had administered naloxone (*p* < 0.001). Those with diagnosed anxiety disorder were also more likely to belong to the high utilization group (*p* = 0.02), although other mental health diagnoses did not differ by utilization group. Lastly, those currently enrolled in opioid agonist therapy were significantly more likely to be assigned to the higher utilization group (*p* = 0.04). We found no differences in substance use patterns by harm reduction practice utilization group.

### Correlation matrix

**Fig. 1:**
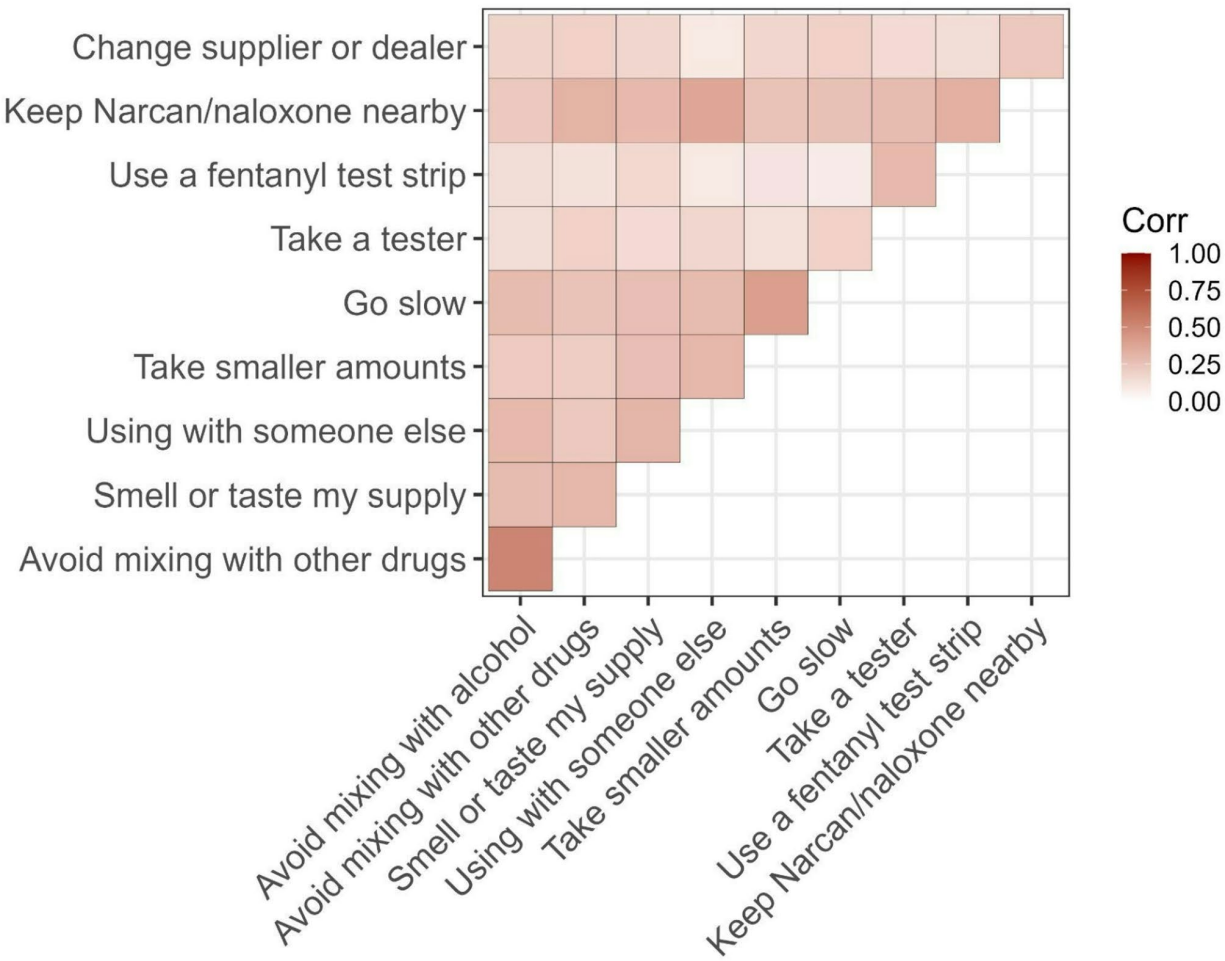
Correlogram of harm reduction practices among 503 people who use drugs in Rhode Island from September 2020 to February 2023

Many participants reported using several harm reduction practices in combination. In pairwise comparisons, some dyads of harm reduction practices were more strongly correlated than others (Fig. 1). In a correlation matrix of harm reduction practices, correlation coefficients ranged from 0.08 to 0.52. The most strongly associated dyads of practices were: “avoid mixing with alcohol” and “avoid mixing with other drugs” (0.52), “go slow” and “take smaller amounts” (0.40), and “use with someone else” and “keep naloxone nearby” (0.37). Notably, “use an FTS” was weakly correlated with “go slow” (0.08) and “using with someone else” (0.09). Correlation coefficients are shown in Supplementary Table 3; statistically significant results are bolded.

## Discussion

We identified no gender-based differences in harm reduction practice typologies (i.e., men and women were proportionally represented across utilization groups). When comparing men and women, there were no differences in the probability of reporting the use of any practice, except that fewer men reported keeping naloxone nearby (*p* = 0.02). This finding is consistent with prior literature documenting gendered labor between men and women who use drugs, with women often playing a greater role in overdose reversal, education, and care [25]. Our findings align with the work of Austin and colleagues, which illustrated that communities of PWUD rely on women to formally and informally support harm reduction efforts, including overdose response [25]. 

Our results show that those belonging to the no/low utilization group were significantly more likely to have a history of incarceration. This finding provides a strong argument to enhance overdose education and naloxone distribution for people—and particularly for men—released from carceral settings. This intervention has been tested extensively [26, 27], as the days and weeks after being released from carceral settings are particularly high periods of overdose risk [28]. Unfortunately, we did not have access to data related to the length of the most recent incarceration or the number of prior incarcerations. Overdose prevention education, especially education utilizing peer-based and theory-based methodologies, should be made available to persons being released from carceral settings to address this gap in harm reduction practice utilization among persons with prior criminal legal system involvement [29]. 

Our finding that those who are single are more likely to be assigned to the no/low utilization group is concerning. These individuals are likely to be at heightened overdose risk because they reported adopting fewer precautionary harm reduction strategies, and may be less likely to have someone available to administer naloxone in the event of an overdose. This finding is supported by prior work from our team, which found heightened overdose death among people who were single [30]. To address the heightened risk of overdose among those who are single and others at risk of overdose due to using alone, jurisdictions should seek to increase access to overdose prevention centers [31, 32], overdose response hotlines [31, 32], and other overdose detection technologies [33].

Those assigned to the high utilization group were more likely to have lifetime experience with IDU, witnessing overdose, performing rescue breathing, and administering naloxone. Prior work has documented that IDU is associated with an elevated risk of nonfatal overdose; [34] thus, it is promising that members of this subgroup at elevated risk of overdose are already engaging in harm reduction practices. Enhancing investment in, and access to, harm reduction strategies, including those mentioned above, may be further protective, both for those who inject and for those who use via other routes of administration.

Notably, “use an FTS” was weakly correlated with “go slow” and “using with someone else.” Previous literature has highlighted that those who use FTS may engage in drug use practices that may or may not reduce overdose risk [35]. At the same time, other work has demonstrated that those who use FTS may lead participants to engage in safer behaviors such as using with someone else and keeping naloxone nearby [36]. FTS are an important harm reduction approach, and harm reduction programs should perhaps strengthen their messaging that FTS are intended to be used alongside other harm reduction strategies. However, our finding that “use an FTS” and “using with someone else” were not correlated suggests that, in the absence of someone to use with, FTS provides an additional safety net. This suggests that FTS use may be particularly beneficial for people who are single and may experience a heightened risk of overdose. In 2018, the state of RI decriminalized FTS [37], and community-based organizations began distributing this resource to people across the state [38]. By the end of the study period in 2023, they were both more accessible and more broadly utilized, yet still less utilized compared to other estimates from other jurisdictions [39]. Although not a primary focus of the present analysis, a recent investigation involving the same cohort found that almost three-quarters of RAPIDS participants had ever heard of FTS, and among this group, half had ever utilized FTS [40]. 

In our cohort of participants who use drugs, 43% reported keeping naloxone nearby as a way to prevent accidental overdose. For context, RI has robust community-based naloxone distribution, with approximately 60,000 kits distributed per year since 2023 [41]. Our findings mirror similar, city-specific estimates of naloxone carriage among PWUD, including recent estimates from Baltimore showing that 42% of participants reported always keeping naloxone nearby when using drugs [42]. The prevalence of naloxone carriage in ours and other samples of PWUD is much higher than recent estimates from the US general population, illustrating that while 71% of adults had heard of naloxone, only 6% reported currently carrying it [43]. 

National momentum to improve access to naloxone at a population level has included standing orders for pharmacy-based naloxone prescribing and the approval of over-the-counter naloxone for use without a prescription [26–28]. However, more targeted interventions to improve naloxone carriage may be necessary. Research has shown that targeted naloxone intervention strategies can be used to improve racial and ethnic inequities [29]. Prior work has also documented more consistent naloxone carriage among women compared to men [30]. This suggests that targeting naloxone dissemination efforts to those not yet fully engaged in other harm reduction practices may be useful. Although many shelters and transitional housing programs have NaloxBoxes in RI [44], it may also be important to provide naloxone training in men-only spaces such as male-focused transitional programs or men’s shelters, or empower men to recognize and respond to the signs of an overdose.

This analysis is subject to some important limitations. It should be noted that differing sample sizes (i.e., only 13% of the sample belonged to the no/low utilization group) may impact significance testing; *p*-values should be interpreted with caution. Self-reporting stigmatized behaviors via interviewer-administered surveys may introduce social desirability bias, particularly as compared to anonymous, computer-based methods. Questions related to the use of fentanyl were operationalized differently than the questions regarding any other substances of interest (i.e., extra-medical prescription stimulants, methamphetamine, heroin), and thus, self-reported past-month fentanyl use was not combined with self-reported past-month heroin use. Although we asked about relationship status and relational patterns of drug use (i.e., helping someone else inject), we did not assess partner drug use or location of use; future work should more deeply interrogate these social and physical contexts of use [45]. Additionally, when investigating further into the “other” harm reduction practices that participants reported (Supplemental Table 5), many practices mentioned either heroin or fentanyl. Still, only one answer mentioned both (‘Don’t use heroin or fentanyl’). In RI, fentanyl has contaminated a wide range of substances (e.g., heroin, crack cocaine, powder cocaine). It is associated with three of every four drug poisoning deaths in the state [46]. Thus, it is most helpful to understand fentanyl use and unintentional fentanyl contamination across a wide range of substances.

Although data regarding gender identities other than men and women are included (Supplementary Table 4), this was not a focus of the study, but it should be analyzed further. Experiences of gender minorities warrant thoughtful and focused research and subsequent programmatic changes. Results may not be generalizable to other jurisdictions, as substance use practices, access to harm reduction-centered resources, and political climate vary substantially by area.

## Conclusions

Results show that women were more likely to keep naloxone nearby and that no other harm reduction practices differed by gender. Although keeping naloxone nearby was the most common harm reduction strategy reported by participants, it is also important to foster other strategies, such as using with someone else, going slower, and using FTS. This analysis can help support pragmatic decision-making about harm reduction-oriented strategies.

## Supplementary Information

Below is the link to the electronic supplementary material.

Supplementary Material 1

## Data Availability

Data may be available from the authors upon reasonable request per correspondence.

## Abbreviations

RAPIDS: Rhode Island Prescription and Illicit Drug Study
US: United States
PWUD: people who use drugs
LCA: latent class analysis
FTS: fentanyl test strips
LGBQ: lesbian, gay, bisexual, and queer
USD: US dollars
IDU: injection drug use
OAT: opioid agonist therapy
AIC: Akaike information criterion
BIC: Bayesian information criterion
