# Supplementary material for: Gender-based differences in harm reduction practices among people who use drugs in Rhode Island: a latent class analysis
Source: Harm Reduct J. 2025 Aug 18;22:140. doi: 10.1186/s12954-025-01295-9 (PMC12359961; doi:10.1186/s12954-025-01295-9)
Supplement: Supplementary file 1 — Supplementary Material 1 [file 12954_2025_1295_MOESM1_ESM.docx]

# Supplementary materials

## Supplementary Table 1. Latent Class Model fit statistics for harm reduction practices among 503 people who use drugs in Rhode Island from September 2020 to February 2023.

|  |  | **Latent Class Size** | | |  |  |
| --- | --- | --- | --- | --- | --- | --- |
| **Fit statistics** | 1 | 2 | 3 | 4 | 5 | 6 |
| Residual degrees of freedom | 491 | 478 | 465 | 452 | 439 | 426 |
| Maximum log likelihood | -3222.78 | -2908.52 | -2798.69 | -2768.03 | -2742.57 | -2726.08 |
| Akaike information criterion (AIC) | 6469.56 | 5867.04 | 5673.37 | 5638.05 | 5613.14 | 5606.17 |
| Bayesian information criterion (BIC) | 6520.20 | 5972.55 | 5833.76 | 5853.50 | 5883.26 | 5931.15 |
| G^2^ likelihood ratio | 1672.25 | 1043.73 | 824.07 | 762.74 | 711.83 | 678.86 |
| Entropy | NaN | 0.76 | 1.00 | 1.00 | 0.99 | 1.00 |

Note: Harm reduction practices included “Avoid mixing with alcohol,” “Avoid mixing with other drugs,” “Smell or taste my supply,” “Using with someone else,” “Take smaller amounts,” “Go slow,” “Take a tester,” “Use fentanyl test trips,” “Keep naloxone nearby,” “Change supplier or dealer,” “Something else,” and “Nothing.”

##

## Supplementary Table 2. Harm reduction practice probabilities of the three-class model among 503 people who use drugs in Rhode Island from September 2020 to February 2023.

|  |  | Latent class probability | | |
| --- | --- | --- | --- | --- |
| Harm reduction practices | | No/low utilization | Moderate utilization | High utilization |
| Avoid mixing with alcohol | | 0.000 | 0.109 | 0.583 |
| Avoid mixing with other drugs | | 0.000 | 0.165 | 0.685 |
| Smell or taste my supply | | 0.000 | 0.078 | 0.535 |
| Using with someone else | | 0.000 | 0.233 | 0.723 |
| Take smaller amounts | | 0.000 | 0.356 | **0.836** |
| Go slow | | 0.016 | 0.296 | **0.795** |
| Take a tester | | 0.000 | 0.180 | 0.445 |
| Use fentanyl test strips | | 0.000 | 0.143 | 0.342 |
| Keep naloxone nearby | | 0.016 | 0.327 | **0.858** |
| Change supplier or dealer | | 0.016 | 0.130 | 0.343 |
| Something else^a^ | | 0.000 | 0.108 | 0.038 |
| Nothing | | **1.000** | 0.000 | 0.007 |

Note:

a) Includes the following responses, among others: stop or avoid drug use, avoid drugs with overdose risk, use the same supplier or talk to supplier, cook or re-cook drugs, use responsibly.

##

## Supplementary Table 3. Correlation coefficients of harm reduction practices among 503 people who use drugs in Rhode Island from September 2020 to February 2023.

| Harm reduction practice 1 | Harm reduction practice 2 | | | | | | | | | |
| --- | --- | --- | --- | --- | --- | --- | --- | --- | --- | --- |
|  | Avoid mixing with alcohol | Avoid mixing with other drugs | Smell or taste my supply | Using with someone else | Take smaller amounts | Go slow | Take a tester | Use fentanyl test strips | Keep naloxone nearby | Change supplier or dealer |
| Avoid mixing with alcohol | 1.00 |  |  |  |  |  |  |  |  |  |
| Avoid mixing with other drugs | **0.521** | 1.00 |  |  |  |  |  |  |  |  |
| Smell or taste my supply | **0.275** | **0.300** | 1.00 |  |  |  |  |  |  |  |
| Using with someone else | **0.287** | **0.233** | **0.308** | 1.00 |  |  |  |  |  |  |
| Take smaller amounts | **0.217** | **0.212** | **0.271** | **0.300** | 1.00 |  |  |  |  |  |
| Go slow | **0.280** | **0.247** | **0.269** | **0.277** | **0.403** | 1.00 |  |  |  |  |
| Take a tester | **0.141** | **0.192** | **0.145** | **0.174** | **0.126** | **0.189** | 1.00 |  |  |  |
| Use fentanyl test strips | **0.137** | **0.120** | **0.156** | 0.085 | **0.107** | 0.078 | **0.289** | 1.00 |  |  |
| Keep naloxone nearby | **0.228** | **0.323** | **0.292** | **0.366** | **0.252** | **0.264** | **0.281** | **0.326** | 1.00 |  |
| Change supplier or dealer | **0.185** | **0.190** | **0.166** | 0.086 | **0.165** | **0.189** | **0.152** | **0.143** | **0.228** | 1.00 |

Note: Bold values denote statistical significance of *p*<0.05.

##

##

## Supplementary Table 4. Cross-tabulation with column percentages showing self-reported current gender identity and biological sex at birth (N=505).

|  |  | **Biological sex at birth** | |
| --- | --- | --- | --- |
|  | **Overall** | **Male**  n=337 | **Female**  n=168 |
| **Current gender identity** |  |  |  |
| Man^a^ | 322 (63.8) | 321 (95.3) | 1 (0.6) |
| Woman^b^ | 166 (32.9) | 3 (0.3) | 163 (97.0) |
| Transgender man (FTM)^a^ | 0 (0.0) | 0 (0.0) | 0 (0.0) |
| Transgender woman (MTF)^b^ | 6 (1.2) | 6 (1.8) | 0 (0.0) |
| Genderqueer^c^ | 3 (0.6) | 2 (0.6) | 1 (0.6) |
| Something else^c^ | 6 (1.2) | 4 (1.2) | 2 (1.2) |
| Don’t know/refused^d^ | 2 (0.4) | 1 (0.3) | 1 (0.6) |

##

##

##

##

##

##

##

##

##

##

##

## Notes:

a) Participants who answered male, transgender man, and female-to-male (FTM) are grouped as men.

b) Participants who answered female, transgender female, and male-to-female (MTF) are grouped as women.

c) Participants who answered genderqueer or something else are grouped as other gender identity.

d) Participants who answered don’t know/refused are excluded from the present analysis.

## Supplementary Table 5. Self-reported harm reduction practices described as “something else” (n=38). Responses that are exactly the same were combined.

| Categorization | Response |
| --- | --- |
| Avoid taking drugs/do not use at all (9) | Avoid taking drugs |
|  | Avoid using |
|  | Not use (2) |
|  | Not use at all (2) |
|  | Stop using (2) |
|  | Stop using altogether |
|  | Try not to use drugs |
| Avoid drugs that I can overdose on (3) | Avoid drugs that I can overdose on |
|  | Doesn't do any drugs that are at risk of overdose |
|  | Don’t use drugs that would cause overdose |
| Do not use specific type(s) of drug(s) (6) | Avoiding heroin |
|  | Don't use heroin |
|  | Don't use heroin or fentanyl |
|  | Don't use heroin/illicit pain pills. |
|  | Stay away from fentanyl/ don't share drug using supplies |
|  | Use non-opioid drugs |
| Routine with dealer (5) | Maintain same dealer |
|  | Same dealer (2) |
|  | Talk to dealer (2) |
| Habits with surrounding people (2) | Avoid being around people who use it |
|  | Watch someone else to see if they have a reaction |
| Cook or recook drugs (2) | Cook it |
|  | Recook it |
| Use responsibly (2) | Be responsible about it |
|  | Use responsible |
| Know body limits (2) | Know body limits |
|  | Stick to usual amount/routine |
| Visual inspection of drugs (2) | If I cook coke, I can tell if it has fentanyl |
|  | Visually inspect drugs for fentanyl |
| Other (5) | Drink water |
|  | Move around more than usual |
|  | Pay attention to signs of overdose (nodding off) |
|  | Try a little bit first |

## 
